# Difficulties in the diagnosis of HbS/beta thalassemia: Really a mild disease?

**DOI:** 10.5937/jomb0-30420

**Published:** 2022-02-02

**Authors:** Süheyl Uçucu, Talha Karabıyık, Fatih Azik

**Affiliations:** 1 Ministry of Public Health Care Laboratory, Department of Medical Biochemistry, Muğla, Turkey; 2 Bursa City Hospital, Department of Medical Biochemistry, Bursa, Turkey; 3 Muğla Sıtkı Koçman University, Faculty of Medicine, Department of Pediatric Hematology-Oncology, Muğla, Turkey

**Keywords:** SCD, sickle cell anemia, HbS/b, HbSS, Sickle-b0-thalassemia, genotype, fenotype, SCD, anemija srpastih ćelija, HbS/b, HbSS, Srp-b0-talasemija, genotip, fenotip

## Abstract

**Background:**

HbS/b cases having clinical, hematologic and electrophoretic similarities cannot be sufficiently distinguished from sickle cell anemia cases and are misdiagnosed as sickle cell anemia. This study will investigate the congruence between the HPLC thalassemia scanning tests and the laboratory findings compared to the DNA sequence analysis results of the patients diagnosed with SCA between 2016 and 2020. This study also aims to indicate the current status to accurately diagnose sickle cell anemia and HbS/b in the light of hematologic, electrophoretic and molecular studies.

**Methods:**

Fourteen patients who were diagnosed with SCA in hospitals at different cities in Turkey and followed by the Thalassemia Diagnosis, Treatment and Research Center, Muğla Sıtkı Koçman University were included in this retrospective study. The socio-demographic characteristics, hemogram, hemoglobin variant analysis results and DNA chain analysis results of the patients were taken from the database of the centre and then examined. The informed consents were taken from the patients. The patients were administered a survey containing questions about transfusion history and diagnostic awareness. The Beta-Thalassemia mutations were analysed using a DNA sequencer (Dade Behring, Germany) based on the Sanger method.

**Results:**

According to the DNA sequence analysis, the results of these patients diagnosed with SCA in hospitals in different cities of Turkey were the following: of 14 patients, 8 had HbS/b0, and HbS/b+ and one had HbS carrier, and one had Hb-O, and three had SCA. The patient with HbS carrier status also contains three additional mutations, all of which are heterozygous. We discovered that although two of three mutations, which are c.315+16G>C and c.316-185C>T, are previously reported as benign, at least one of the two mentioned mutations, when combined with HbS, causes transfusion-dependent HbS/b.

**Conclusions:**

Briefly, HbSS and HbS/b thalassemia genotypes cannot be definitely characterized by electrophoretic and hematologic data, resulting in misdiagnosis. c.315+16G>C and c.316-185C>T are previously reported as benign; at least one of the two mentioned mutations, when combined with HbS, causes transfusion-dependent HbS/b. In undeveloped or some developing countries, molecular diagnosis methods and genetic analyses cannot be used. If mutation analyses could be performed, then such differential diagnosis errors would reduce. However, if mutation analysis cannot be performed, other methods such as HPLC, capillary electrophoresis absolutely be sought to have insight into the parental carriage status.

## Abstract (sr)

**Uvod:**

Slučajevi HbS-b koji imaju kliničke, hematološke i elektroforetske sličnosti ne mogu se dovoljno razlikovati od slučajeva anemije srpastih ćelija i pogrešno se dijagnostikuju kao anemija srpastih ćelija. Ova studija će istražiti podudarnost između HPLC testova za skeniranje talasemije i laboratorijskih nalaza u poređenju sa rezultatima analize DNK sekvence pacijenata sa SCA dijagnozom između 2016. i 2020. Ova studija takođe ima za cilj da ukaže na trenutni status tačne dijagnoze anemije srpastih ćelija i HbSu svetlu hematoloških, elektroforetskih i molekularnih studija.

**Metode:**

U ovu retrospektivnu studiju je bilo uključeno četrnaest pacijenata kojima je dijagnostikovan SCA u bolnicama u različitim gradovima Turske, a koje je pratio Centar za dijagnozu, lečenje i istraživanje talasemije, na Univerzitetu Muğla Sitki Kočman (Muğla Sıtkı Koçman). Socio-demografske karakteristike, hemogram, rezultati analize varijante hemoglobina i rezultati analize DNK lanca pacijenata su uzeti iz baze podataka centra i potom ispitani. Od pacijenata je obezbeđena informisana saglasnost. Pacijentima je data anketa koja je sadržala pitanja o istoriji transfuzije i svesti o dijagnozama. Mutacije beta-talasemije su analizirane pomoću DNK sekvencera (Dade Bering, Nemačka) na osnovu Sangerove metode.

**Rezultati:**

Prema rezultatima analize DNK sekvence ovih pacijenata kojima je dijagnostikovan SCA u bolnicama u različitim gradovima Turske od 14 pacijenata je 8 imalo HbS-b0 i HbS-b+, a jedan je bio HbS nosilac, jedan HbO, a tri osobe su imale SCA. Pacijent sa statusom nosioca HbS je takođe imao tri dodatne mutacije koje su sve heterozigotne. Otkrili smo da, iako su dve od tri mutacije, a to su c.315+16G>C i c.316-185C>T, prethodno prijavljene kao benigne, bar jedna od dve pomenute mutacije, u kombinaciji sa HbS, izaziva transfuziono zavisni HbS/b.

**Zaključak:**

Ukratko, genotipovi HbSS i HbS/b talasemije ne mogu se definitivno okarakterisati elektroforetskim i hematološkim podacima, što rezultira pogrešnom dijagnozom. Utvrđeno je da bar jedna od dve pomenute mutacije, c.315+16G>C i c.316-185C>T, koje su prethodno prijavljene kao benigne, u kombinaciji sa HbS izaziva transfuziono zavisni HbS/b. U nerazvijenim zemljama, ili nekim zemljama u razvoju, metode molekularne dijagnoze i genetske analize se ne mogu koristiti. Ako bi se mogle izvršiti analize mutacija, onda bi se takve greške diferencijalne dijagnoze smanjile. Međutim, ako se analiza mutacija ne može izvršiti, apsolutno se traže druge metode poput HPLC-a, kapilarne elektroforeze kako bi se dobio uvid u status prenošenja sa roditelja na decu.

## Introduction

Sickle cell diseases (SCD) affect millions of people worldwide. It is estimated that there are more than 300 million people who have (HbSS, SC, SD, SE, S/β, SO-Arab) or carry sickle cell disease, which is expected to be increased gradually [1] [2]. 300,000-400,000 babies with SCA are born every year around the world, and tens of thousands of people have the most severe clinical phenotype of the disease - the homozygous HbSS form [3] [4]. It can be seen everywhere in the world, but it has an epidemic course, especially in Sub-Saharan Africa, Saudi Arabia, India, Central and South America, Middle East countries and Mediterranean countries [5] [6]. In Turkey, sickle cell disease and carriage are common, especially in the southern Mediterranean coasts, with a prevalence ranging from 0.3% to 44% [7].

Sickle cell disease is the most common one of the monogenic disorders caused by a point mutation in the 6th codon of the β-globin gene (HbS or HbB: c.20A> T) [6] [8]. It originates from homozygous or compound heterozygous mutation of the abnormal hemoglobin S that forms as a result of the conversion of glutamic acid in the β-globin chain to valine [6] [9]. SCD has a multi-systematic and complex physiopathology that can damage every organ and tissue in the body [8]. The trigger of all complications is the deoxygenated HbS polymerization [6]. The genotype is a key determinant of the clinical severity of SCD [4] [10].

More than 15 genotypes, which cause sickle cell disease, were determined [11]. Homozygosis is the most common and severe genotype of the disease with the shortest survival and is called sickle cell anemia (SCA) [4] [11]. Other main pair heterozygote SCD types are the hemoglobin S/DPunjab, hemoglobin S/E, hemoglobin SO-Arab, hemoglobin SC disease (HbS/C), sickle-β +-thalassemia (HbS/β+) and sickle-β^0^-thalassaemia (HbS/β^0^) forms. HbS/β cases are called HbS/β+ and HbS/β^0^ according to the different hemoglobin A levels. Higher hemoglobin A and F levels are characterized by a milder phenotype. It is difficult to clinically distinguish S/β^0^ thalassemia from sickle cell anemia [4] [8] [10]. Therefore, parenteral carriage status and mutation should be analysed for an accurate diagnosis [4] [10].

In particular, HbS/β cases having clinical, hematologic and electrophoretic similarities cannot be sufficiently distinguished from sickle cell anemia cases, and are misdiagnosed as sickle cell anemia [10]. Although they are clinically treated similarly, the specific profiles of the genetic and pathophysiological mechanisms of patients with HbS/β are not yet well known. On the other hand, there are limited data to describe the profile of clinical complications of HbS/β patients [8]. The clinical phenotype severity varies even among individuals with the same genotype [12]. The complication incidence varies over time in the same individuals and among different individuals [4] [11]. Patients manifest a dramatic range of severity ranging from a milder clinical course to severe transfusion dependence and progressive organ damages [8].

One of the important points is that SCA manifests itself in the second 6-month postpartum period, while HbS/β may not manifest until puberty [7]. However, it should be noted that even patients with mild SCD (HbSC and HbSβ+) forms may have vasoocclusion attacks and hemolytic anemia as well as all serious and life-threatening complications, which are seen in SCA [13] [14]. If a patient with HbS/β is misdiagnosed as SCA, this would have significant effects for the next generation; hence an accurate diagnosis is crucial to prevent future diseases for the next generations.

This study will retrospectively investigate the congruence between the HPLC thalassemia screening tests and the laboratory findings in comparison with the DNA sequence analysis results to understand how accurately patients with SCA were diagnosed based on this information.

## Materials and Methods

Ten (73.4%) female and four (26.6%) male patients, who admitted to the Thalassemia Diagnosis, Treatment and Research Centre, Muğla Sıtkı Koçman University Education and Research Hospital between January 1, 2016, and October 31, 2020, were included in this retrospective study. The ethics approval was taken from Muğla Sıtkı Koçman University Education and Research Hospital on July 3, 2020, and the Ethics Committee on September 26, 2020, with the document numbered 2020/10. This study was conducted following the Helsinki Declaration's principles.

Fourteen patients who were diagnosed with SCA in hospitals in different cities in Turkey and followed by the Thalassemia Diagnosis, Treatment and Research Centre, Muğla Sıtkı Koçman University were included in the study. The socio-demographic characteristics, hemogram, hemoglobin variant analysis results and DNA chain analysis results of the patients were taken from the database of the centre and then examined. The informed consents were taken from the patients. The patients were administered a survey containing questions about transfusion history and diagnostic awareness.

Their red blood cell index parameters were determined using Sysmex XN 1100 (Sysmex Diagnostic, Japan).

The hemoglobin variant analysis was performed using Primus Ultra II device (Trinity Biotech Diagnostic, Ireland) based on the high-pressure liquid chromatography (HPLC) byion exchange chromatography.

The Beta-Thalassemia mutations were analysed using a DNA sequencer (Dade Behring, Germany) based on the Sanger method.

## Results

Our patient group consisted of 10 female (73.4%) and 4 male (26.6%) patients aged 6 months to 54. The main findings of the study; our patients had a wide range of clinical severity, ranging from mild joint pains to transfusion dependence.

According to the DNA sequence analysis, the results of these patients diagnosed with SCA in hospitals in different cities of Turkey were the following: of 14 patients, 8 had HbS/β^0^ and HbS/β+ and one had HbS carrier, and one had Hb-O, and three had SCA.

The patient with HbS carrier status also contains three additional mutations, all of which are heterozygous. Although two of three mutations, which are c.315+16G>C and c.316-185C>T, are previously reported as benign, at least one of the two mentioned mutations, when combined with HbS, causes HbS/β status [15]. This patient is transfusion-dependent and has pain crises. This patient's MCV value indicates microcytosis, which is compatible with HbS/β.

The patients in our study had the first manifestations and the initial diagnosis at 5, 6 or 12 years old.

## Discussion

This study will investigate the congruence between the HPLC thalassemia scanning tests and the laboratory findings in comparison with the DNA sequence analysis results of the patients diagnosed with SCA between 2016 and 2020. This study also aims to indicate the current status to accurately diagnose sickle cell anemia and HbS/β in the light of hematologic, electrophoretic and molecular studies (Table 1).

**Table 1:** Hematologic differences between SCA and S/Beta in literature

Patients & Tests	HbSS	Hb S/β0	Hb S/β+
RBC Morphology	Normocytic	Microcytic	Microcytic
	Normochromic	Microchromic	Microchromic
Hemoglobin Electrophoresis			
A2 (%)	<3.5	>3.5	>3.5
F (%)	<10	<20	<20
A0 (%)	0	0	20–30
S (%)	>90	>80	>60
Phenotype	Usually Heavy	Medium-Heavy	Mild-Medium

In the literature, HbS/β was characterized by fewer sickle cells and microcytic, hypochromic RBC, and a distinction was made between normocytic normochromic anemia and SCA to describe the explanatory variables between hemoglobin HbSS and HbS/β (Table 2) [5] [6]. However, splenomegaly is an important distinctive finding among the clinical symptoms. It was reported that SCA patients have hemolytic anemia with a progressive course in the second 6-month postpartum period and early childhood period as well as chronic splenomegaly, acute splenic sequestration crisis, splenic infarctions. However, HbS/β patients have no splenic infarction in the childhood period but have severe, palpable splenomegaly and associated sequestration crisis and rarely organ failure in the adulthood period [16] [17]. In the multi-centre study by Belgemen Özer et al. [18], the hematologic, molecular and clinical data of 55 HbS/β patients were analyzed and compared with the literature data. It was reported that there were cases that were not consistent with the literature, with varying hemograms, hemoglobin electrophoresis and peripheral smear findings. In the study performed by Benites et al. [19] to compare the hematologic parameters of HbS/β^0^ and HbS/β+ patients, a statistically significant difference was not found in any hemogram parameter except for leukocytes and platelets.

**Table 2:** Hematologic and molecular diagnostic data of patients with SCA Note:*RBC, red blood cell; Hb, hemoglobin concentration; MCV, mean cell volume; MCH, mean corpuscular hemoglobin

Pt.	Age	Sex	RBC (10^12cells/L)	HGB (g/L)	MCV (10^-14 L/cells	MCH (10^-11 g/cells)	B12 (pmol/L)	Folic Acid (nmol/L)	Hb S (%)	Hb A2 (%)	Hb A0 (%)	Hb F (%)	Genotype (Only pathogenic variants shown)	Phenotype
1	19	M	2.82	86	8.76	3.05	231	> 45	61.7	4.5	10.7	28	-	Hb S/β
2	38	F	3.69	84	7.05	2.28	312	24	46.4	5.8	46.2	1.6	heterozygous c.20A>T, heterozygous c.25_26delAA	Hb S/β0
3	40	F	3.46	91	7.80	2.63	188	> 45	44.9	5.4	44.2	5.5	heterozygous c.20A>T	Hb S Carrier
4	35	F	2.84	65	7.15	2.29	154	17	50.8	5.5	40.7	3.0	heterozygous c.20A>T, heterozygous c.316-106C>G	Hb S/β+
5	35	F	2.06	76	10.19	3.69	364	15	69.9	4.9	20.3	4.9	homozygous c.20A>T	Hb SS
6	54	M	5.48	120	6.55	2.19	-	-	-	5.3	14.9	1.8	heterozygous c.93-21 G>A, heterozygous c.364G>A	Hb O-Arab/β+
7	38	F	3.37	74	6.71	2.20	-	-	72.5	6.1	13.2	8.2	herterozygousc.20A>T, heterozygous c.93-21G>A	Hb S/β+
8	24	F	2.55	59	7.37	2.31	-	17	76.7	6.9	9.0	7.4	heterozygous c.20A>T, heterozygous c.316-106C>G	Hb S/β+
9	42	M	2.84	86	8.59	3.03	441	39	82.2	4.9	2.9	10	homozygous c.20A>T	Hb SS
10	60	F	2.19	85	11.05	3.88	242	18	48.3	5.2	4.0	42.5	heterozygous c.315+1G>A, heterozygous c.20A>T	Hb S/β0
11	54	F	2.72	53	5.90	1.95	352	-	82.8	6.1	3.4	7.7	-	Hb S/β
12	26	M	3.45	96	7.68	2.78	245	12	77	3.7	4.6	14.7	homozygous c.20A>T	Hb SS
13	30	F	2.61	100	9.89	3.83	-	-	78.5	4.7	3.0	13.8	homozygous c.20A>T	Hb SS
14	18	F	3.79	92	7.28	2.43	-	-	69.5	6.5	11.4	12.6	heterozygous c.20A>T, heterozygous c.93-21G>A	Hb S/β+

The results of our study on this controversial subject are mostly compatible with the literature. However, in analogy to the studies by Benites et al. [19] and Belgemen Özer et al. [18], our study had also patients who were incompatible with the literature. A HbSS patient had a low MCV; however, an elevated MCV was noted in some HbS/β patients, but it was found that this was caused by megaloblastic anemia. MCV/MCH is the basic distinctive hemogram finding between the two diseases; however, it is not sufficiently distinctive in cases of iron deficiency, B12-folic acid deficiency, nutritional deficiency, hypothyroidism, sider oblastic anemia, myelodysplastic conditions, HbSS accompanied by alpha-thalassemia mutation. Iron deficiency may cause the interpretation of HbSS as HbS/β, while megaloblastic anemia may cause the interpretation of HbS/β as Hb SS. Overlooking this may lead to erroneous diagnosis. However, it should be noted that it may be rarely detected at different values [20] [21].

In the study by Notarangelo et al. [12], HbS/β patients with confirmed molecular accuracy were classified according to the β mutation, and it was reported that some mutation differences with IVS-I-5 (G>C), IVS-I-5 (G>A) and IVS-I-110 had a more severe phenotype and clinical presentation, and some mutation differences were associated with a milder phenotype [8]. The study by Belisário et al. [22] reported that the newly discovered 92 (C>T) and IVS-II-844 (C>A)/IVS-II-839 (T>C) mutation presented as a very mild HbS/β+ case.

The patient with HbS carrier status also contains three additional mutations, all of which are heterozygous. We discovered that although two of three mutations, which are c.315+16G>C and c.316-185C>T, are previously reported as benign, at least one of the two mentioned mutations, when combined with HbS, causes HbS/β status. This patient is transfusiondependent and has pain crises [15]. This patient's MCV value indicates microcytosis which is compatible with HbS/β. Especially in heterozygote individuals, the relationship between genotype and phenotype is important since it significantly affects the clinical severity. Some mutation differences are associated with milder phenotype, while some are associated with severe and progressive organ damages and predictable complications [23].

When examining the congruence between HPLC and DNA sequence analysis, patients in the study received transfusion regularly and used hydroxyurea. Therefore, hemoglobin A0, A2, F and S values may be misleading. However, the DNA sequence analysis results are the final diagnostic for HbS/β. On the other hand, a HbA2>3.5 is interpreted in favour of HbS/β, while a HbA2<3.5 is interpreted in favour of SCA, in the literature. In the commonly used hemoglobin electrophoresis, since electrophoretic migrations and elution patterns of HbS and HbA2 arethe same, they migrate together, and many abnormal hemoglobin values coincide at small values. Therefore, due to a methodological error in electrophoresis, the HbS band sometimes erroneously moves the HbA2 band over itself so that it seems to be high, or a part of the HbS band overlaps with the HbA2 band so that the HbA2 band seems to be higher than as is, eventually leading to diagnostic errors. Due to these errors, the normal HbA2 seems to belowered so that accompanying alpha thalassemia can be assumed mistakenly, or the normal HbA2 seems to be elevated so that it can be classified as HbS/β. Similarly, the presence of paraprotein or a high concentration of polyclonal immunoglobulin may cause different hemoglobin band errors [5] [24] [25].

Although HPLC has more advantages, it should be noted that lowered HbA2 values may be measured due to errors caused by the co-elution of HbS and HbA2, analytic references, alpha thalassemia, iron deficiency or delta gene mutations [5]. Electrophoresis has been commonly performed, but HPLC and DNA mutation analyses could not be performed in the past. However, today opportunities increase with advancing technology. Parenteral screening, molecular diagnosis methods and genetic consultation are recommended to make the final diagnosis, clarify the genotype-phenotype correlation and improve the predictability of complications.

The survey with patients in our study revealed that although the majority of patients are young, they do not know the nature of the disease and that parenteral screening was not performed for most patients in the diagnosis period. However, it is remarkable that the patients stated that they had known the two diseases. As is seen in the patients' statements in Table 3, some patients were misdiagnosed and received the wrong treatment until very old age. It was found that the majority of the patients were diagnosed with SCA, but they had S/Beta. In our study, a patient had a major diagnosis of Betathalassemia since infancy, but it was found that she/he really had HbS/β when she/he was 36 years old. Similarly, the study by Eröz et al. [26] found that the patient aged 36 who was diagnosed with sickle cell anemia had beta-thalassemia based on the parenteral screening and mutation analysis results.

**Table 3:** Case histories

Pt.	Diagnosis	Age at first diagnosis and first complaint	DNA sequencing at first diagnosis	Investigation of carrier status in parents at first diagnosis	Transfusion frequency	Splenectomy
1	BT Major At age 4: Hb S/β	Infancy. Unknown complaint	No	Yes.<br>Unknown by the patient	Every 3 weeks	No
2	SCA and BTI At age 36: Hb S/β	5 years. Unknown complaint	No	No.<br>Done at age 36	Every few months from five years of age	Yes
3	SCA and BTI At a later age: Hb S/β	Between 6–12 months. Jaundice	No	No.<br>Done at a later age	Every 4–5 months	Yes
4	BT Major At age 17: Hb S/β	1.5 years. Unknown complaint	Yes	Yes.<br>Carrier sibling	Every 2–4 months	Yes
5	At age 5: SCA At age 35: BTI At a later age: Hb S/β	5 years. Abdominal bloating and bone pain	Yes	No.<br>Carrier sibling	Once a year	No
6	SCA	4–5 years. Fever and severe abdominal pain	No	No.<br>Carrier sibling	Just once	No
7	Hb S/β	2 years. Crisis	No	Yes.<br>Carrier sibling	Once or twice a year	No
8	At age 1.5: SCA At age 14: Hb S/β	1.5 years. Flu	No	Yes.<br>Carrier sibling	Monthly	No
9	Hb S/β	5 years. Joint pain	No	Yes.<br>Carrier sibling	Ten times a year	No
10	SCA	12 years. Never-ending pain crisis	No	No.<br>Unknown by the patient	Twice a year	Yes
11	Hb S/β	6 years. Pains	No	Yes.<br>Carrier sibling	Just once exchange transfusion	No
12	N/A	N/A	N/A	N/A	N/A	N/A
13	SCA	6 months. Unknown complaint	No	No.<br>Affected sibling	3–4 times a year	No
14	SCA	2 years. Pain in joints, arms and feet	No	Yes.<br>Three siblings with SCA	Once a year<br>(First one this year)	Yes

One of the important points is that SCA manifests itself in the second 6-month postpartum period, while HbS/β may not manifest until puberty [7]. Given that SCD is a multi-systematic disease that may damage every organ and tissue in the body and has a complex pathophysiology, it may present with a wide range of clinical complications and organ damages. It may cause serious and life-threatening complications [13] [14]. Patients who were admitted to a hospital with complaints of stroke and sequestration were reported [12] [27]. Sequestration crisis is seen in HbSS or HbSβ^0^ cases before the age of 5, while, in HbS/β cases, it may delay until puberty and early adulthood and progress rapidly, resulting in death as a result of hypovolemic shock [12].

Consequently, it was found that hematologic and electrophoretic studies cannot sufficiently distinguish between the two conditions, causing wrong diagnoses. Although epidemiological and laboratory data are well known, and molecular analyses are more accessible today, it should be noted that data may be overlooked and mistaken. For pediatric patients, it may be live-saving to identify children with HbSS and HbS/β thalassemia, to have information about the genotype, to estimate progressive organ damages and complications in patients with a more severe phenotype, to organize parent training to recognize the symptoms and to plan specific supervision, care and monitoring [28]. The need for transfusion may be high due to severe splenomegaly, which is more commonly seen, especially in HbS/β patients. This transfusion need may be reduced after splenectomy [29] [30].

Also, if a patient with HbS/β is misdiagnosed as SCA, this would have significant effects for the next generation; hence an accurate diagnosis is a critical initial step to prevent future diseases for the next generations [24].

## Conclusion

Briefly, HbSS and HbS/β thalassemia genotypes cannot be definitely characterized by electrophoretic and hematologic data, resulting in misdiagnosis. In undeveloped or some developing countries, molecular diagnosis methods and genetic analyses cannot be used. If mutation analyses could be performed, then such differential diagnosis errors would reduce. However, if mutation analysis cannot be performed, other methods such as HPLC, capillary electrophoresis should be sought to have insight into the parental carriage status.

## Dodatak

### Limitations of the study

The main limitation of this study was the small number of patients in our study population. The patients have received transfusion regularly, and we could not reach the DNA results of two patients. However, it provides an insight into HbSS-HbS/β in Turkey. To clarify this situation, the number of patients may be increased, and sex differences may be examined and analysed, which may fill a gap in the general population and affect the control of the disease.

### Conflict of interest statement

All the authors declare that they have no conflict of interest in this work.
